# Accumulation of Particles and Formation of a Dissipative Structure in a Nonequilibrium Bath

**DOI:** 10.3390/e24020189

**Published:** 2022-01-27

**Authors:** Steven Yuvan, Martin Bier

**Affiliations:** 1 Department of Physics, East Carolina University, Greenville, NC 27858, USA; yuvans16@students.ecu.edu; 2 Faculty of Mechanical Engineering, Institute of Mathematics and Physics, University of Technology and Life Sciences, 85-796 Bydgoszcz, Poland

**Keywords:** Lévy noise, nonequilibrium thermodynamics, active particles, entropy production, dissipative structures

## Abstract

The standard textbooks contain good explanations of how and why equilibrium thermodynamics emerges in a reservoir with particles that are subjected to Gaussian noise. However, in systems that convert or transport energy, the noise is often not Gaussian. Instead, displacements exhibit an α-stable distribution. Such noise is commonly called Lévy noise. With such noise, we see a thermodynamics that deviates from what traditional equilibrium theory stipulates. In addition, with particles that can propel themselves, so-called active particles, we find that the rules of equilibrium thermodynamics no longer apply. No general nonequilibrium thermodynamic theory is available and understanding is often ad hoc. We study a system with overdamped particles that are subjected to Lévy noise. We pick a system with a geometry that leads to concise formulae to describe the accumulation of particles in a cavity. The nonhomogeneous distribution of particles can be seen as a dissipative structure, i.e., a lower-entropy steady state that allows for throughput of energy and concurrent production of entropy. After the mechanism that maintains nonequilibrium is switched off, the relaxation back to homogeneity represents an increase in entropy and a decrease of free energy. For our setup we can analytically connect the nonequilibrium noise and active particle behavior to entropy decrease and energy buildup with simple and intuitive formulae.

## 1. Introduction

The thermodynamics and statistical physics of particles at equilibrium is a standard part of the undergraduate curriculum. The First and Second Law of Thermodynamics are powerful concepts that lead the way to the explanation of many real-life phenomena. Further development led to notions such as the Boltzmann Distribution, the Fluctuation-Dissipation Theorem, Onsager’s Reciprocal Relation, and Microscopic Reversibility [1]. Even setups that are close-to-equilibrium can often be successfully analyzed with these ideas. No general theory, however, is available for systems that are far-from-equilibrium. None of the above laws and notions apply in that case.

Imagine a liquid in which “active” particles are suspended. Such “active” particles can be bacteria that propel themselves, i.e., swim. These can also be particles that are manipulated through fields from the outside. Obviously, energy is pumped into such systems and no First Law or any of the concepts mentioned in the previous paragraph applies. Over the last two decades, setups with active particles have been the subject of much experimental and theoretical research.

There are many different ways to model the movements of active particles. One can, for instance, assume that the particle has the same speed all the time and that the change of the direction of motion follows a diffusion equation [2]. The “Run-and-Tumble” model is a more discrete version of this and it is inspired by the way that *Escherichia coli* bacteria move [3]. Here the particle or bacteria covers a finite-length straight segment at a constant speed. After coming to a stop, it lingers for a moment. It “tumbles” and then picks a new random direction for the next run. There are also different ways to let the active particle interact with the wall of the reservoir in which it swims.

**Figure 1:**
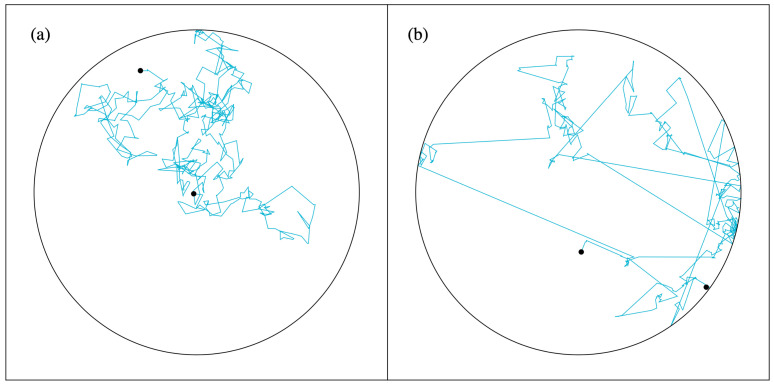
Random walk in a circular domain. Whenever the particle hits the wall, it comes to a standstill and later only moves again when a computed step leads to a movement inside the circle. For every step, the direction is picked randomly and the displacement is drawn from a (**a**) Gaussian distribution or from a (**b**) Lévy-stable distribution. The circle has a radius of 20. Both distributions are symmetric around zero. The Gaussian distribution has a standard deviation of $\sqrt{2}$. For the Lévy-stable distribution, we have α=1 and a scale factor of σ=1.

In our analysis below, we focus on the 2D random walk: At every timestep, a direction is picked randomly and a displacement is drawn from a zero-centered distribution (cf. Figure 1). We let the random walks happen in a confinement. Whenever the particle hits the wall, it comes to a standstill. Subsequently, it only moves away from the wall again if a random displacement makes it move inside the circular confinement.

If displacements are drawn from a zero-average Gaussian distribution, we eventually see a homogeneous distribution of particle positions over the entire domain. However, if we instead draw distances from a so-called α-stable distribution (sometimes called a Lévy-stable distribution) [4,5,6,7], a nonhomogeneous distribution develops.

The Gaussian distribution has an exponential tail, i.e., $p_{2}\left(\xi \right)\propto \exp\left[-\xi^{2}/2\sigma^{2}\right]$ as ξ→±∞. Here σ denotes the standard deviation of the Gaussian. The rapid convergence to zero of the exponential tail means that the probability to make a big jump is very small and effectively negligible. Figure 1a shows this clearly.

For an α-stable distribution, the asymptotic behavior is described by a power law:

(1) $$p_{\alpha }\left(\xi \right)\propto {\left|\xi \right|}^{-\alpha -1}\;\;\mathrm{as}\;\;\xi \to \pm \infty .$$

Here α is the so-called stability index for which we have 0<α<2. For α=2, the Gaussian is re-obtained. The power law converges slower than the exponential. A result of this is that outliers, i.e., large “Lévy jumps”, regularly occur (see Figure 1b). Ultimately, the Lévy walk resembles a run-and-tumble walk, but, following Equation (1), the Lévy jumps have no characteristic length and the average length of a Lévy jump actually diverges.

The Central Limit Theorem [8] tells us that the Gaussian distribution is what ensues when an outcome is the result of multiple stochastic inputs. However, the theorem only applies if all of the constituent stochastic inputs have a *finite* standard deviation. For stochastic inputs with infinite standard deviations, the α-stable distribution is what results.

The α-stable distribution is a standard feature of the *Mathematica* software package and the programming for a simulation as the one leading to Figure 1b is a matter of just a few lines of code. The probability density of the α-stable distribution is given by a big and cumbersome formula [9] and we will not elaborate on it.

Alpha-stable distributions do not just provide a good model for the behavior of active particles. It turns out that power-law tails commonly occur in systems that are far-from-equilibrium with no active particles involved. Almost 60 years ago, Benoit Mandelbrot discovered that variations in the price of cotton futures follow a distribution with an α=1.7 power-law tail [10,11]. More power-law tails and α-stable distributions were identified in the 1990s [12,13,14] when desktop computers became available that could rapidly and easily perform the necessary data processing. As of yet, there is no complete and general theory to explain how and why α-stable distributions are connected to far-from-equilibrium. In this sense, the α-stable distributions are like 1/f-noise [11,15]. The connection of far-from-equilibrium with α-stable distributions and 1/f-noise is still for the most part, a phenomenological one.

Nevertheless, as mentioned above, nonequilibrium characteristics do emerge when, instead of Gaussian noise, Lévy noise is added to particle dynamics. Take a particle doing Brownian motion on a potential V(x). Microscopic reversibility means that every trajectory x(t) from an initial position $x_{i}$ to a final position $x_{f}$ and taking a time Δt, is traversed equally often in forward and backward direction. Microscopic reversibility is an equilibrium feature that is implied by the fact that there can be no arrow of time for a system at equilibrium, i.e., there must be time-reversal symmetry. In 1953, Onsager and Machlup gave mathematical rigor to this idea when they proved that with Gaussian noise, the most likely trajectory up a potential barrier is the reverse of the most likely trajectory down that same barrier [16,17,18]. It can also be rigorously proven that for Lévy noise, the most likely trajectory up a potential barrier is not the reverse of the most likely trajectory down a potential slope [19]. The presence of Lévy noise breaks the time-reversal symmetry that is implicit in equilibrium [20].

**Figure 2:**
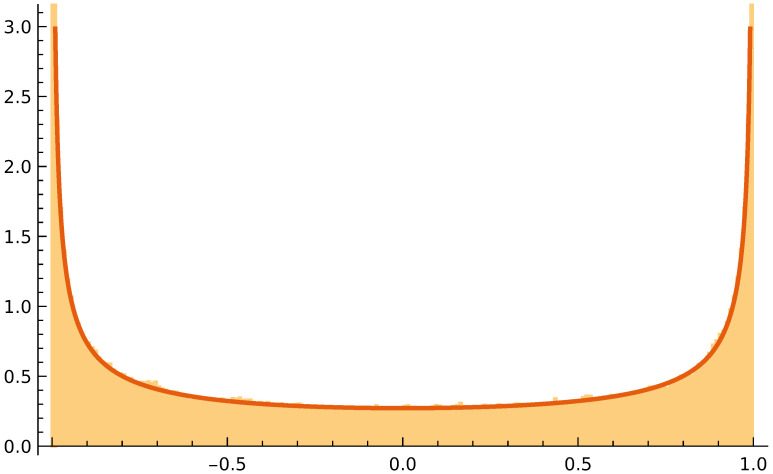
A Lévy walk on the interval −1≤x≤1 (cf. Equation (3)). The value of the stability index is α=0.8. Whenever the particle hits x=±1, it stays there until an iteration occurs in the direction away from the wall. The red curve shows the analytic solution (cf. Equation (4)). The normalized histogram is the result of a numerical simulation of Equation (3); the timestep was Δt=0.001, there were $10^{7}$ iterations, and the scale factor of the symmetric, zero-centered Lévy distribution was taken to be one.

For the setup that is depicted in Figure 1b, the violation of time-reversal symmetry is in the interaction of the particle with the wall. Elastic collisions have time-reversal symmetry and had we taken the particle in Figure 1b to collide elastically with the wall, forward and backward trajectories would have been indistinguishable. Lévy jumps are rare, but because of their length, they are likely to end at the wall. Once the particle is located at the wall, the probability that the first subsequent step is already a Lévy jump away from the wall is small. Moreover, only a step that leads to a movement inside the reservoir will be processed in the simulation. Thus, the particle can “linger” near the wall after hitting it. In the end it appears as if it is easier to get to the wall than it is to get away from it, i.e., it looks as if there is reduced mobility near the wall. Figure 2 shows how this is the case on a 1D interval.

In the previous paragraph, we put the finger on something that applies generally for active particles in a confinement. They do not distribute homogeneously, but instead accumulate near a wall. It furthermore appears that the accumulation is stronger if the wall has a stronger inward curvature [21]. Active particles tend to get stuck in nooks and corners of a confinement and even more so if the nooks and corners are tighter. This is the phenomenon that we will elaborate on below.

The way Lévy particles distribute on a confined 1D segment (cf. Figure 2) can be described with a Fractional Fokker–Planck Equation [22]. The steady-state solution of that equation is available [23]. We show in Appendix A how this solution readily generalizes to higher dimensional setups. Below we examine how Lévy particles distribute over two connected reservoirs where one reservoir is a scaled down version of the other. We will see a deviation from the homogeneous distribution that is obtained when the noise is Gaussian and when equilibrium theory applies.

Suppose we have a volume *V* with *N* indistinguishable particles in it. We partition the initially empty *V* into two reservoirs of a volume V/2 each. Next the particles are inserted. Each reservoir has a probability of 1/2 to receive each particle. Eventually, the probability for all particles to end up in one particular reservoir is $2^{-N}$. The probability for an equal distribution over the two reservoirs is NN/22−N. The binomial coefficient NN/2 grows very rapidly with *N*.

The reason that the air in a room never spontaneously concentrates in one half of the room is that there is just one way to put all molecules in one chosen half and NN/2 ways to distribute them equally. In other words, the macrostate in which all air is concentrated in one particular half of the room has *one microstate* and the macrostate with a homogeneous air distribution over the entire room has NN/2 *microstates*. The entropy of a macrostate can be defined as a scalar value that is proportional to the logarithm of the number of microstates of that macrostate [1]. In this case, it is obvious that the homogeneous distribution leads to maximal entropy.

With a partition and a pump it is, of course, possible to bring all of the air molecules to one half of the room. Such a process requires energy and with standard thermodynamics, the involved energies can be calculated. That energy-consuming, active particles can accumulate in a smaller subvolume does not violate laws of nature, and it is also possible to calculate the entropy change associated with such accumulation. We will perform such a calculation.

The ultimate goal would be a Lévy-noise-equivalent of entropy. This would be a quantity that takes its extreme value when Lévy-noise-subjected particles reach a steady state distribution. The Kullback–Leibler divergence [24] is a positive scalar value that can be thought of as a “distance” between two given distributions. The Kullback–Leibler divergence between the steady state distribution and another distribution could be a good candidate. With tools like Noether’s Theorem, alternative formulations of active-particle statistical mechanics and of the Fractional Fokker–Planck Equation have been derived [25,26], with work in this direction appearing to be promising.

No general formalism is developed in this article, but we present a setup where the entropy decrease associated with the accumulation can be readily described with simple and intuitive formulae. The nonhomogeneous steady-state distributions that develop in the presence of nonequilibrium noise can be interpreted as dissipative structures [27]. The deviation from homogeneity decreases the entropy. However, active particles pump energy into the system and the dissipative structure ultimately facilitates a steady-state dissipation of energy and production of entropy.

## 2. The 1D and 2D Random Walk in a Confined Domain

For a particle in 1D, Brownian motion is commonly described with a Langevin equation:

(2) $$\dot{x}\left(t\right)=\sqrt{2D}\;\xi_{2}\left(t\right),$$

where x(t) is the time-dependent position of the particle, ${}^{•}\equiv d/dt$, *D* is the diffusion coefficient, and $\xi_{2}\left(t\right)$ is a stochastic function that describes the effect of collisions with molecules in the medium. To account for the effect of such collisions, a random number $\theta_{2,i}$ is drawn at the *i*-th timestep. In a simulation with finite timesteps of Δt, we then take $\Delta x_{i}=\sqrt{2D}\;\xi_{2,i}\left(t_{i}\right)\;\Delta t=\sqrt{2D\Delta t}\;\theta_{2,i}$. If Δt is large enough to contain a significant number of collisions, then the aforementioned Central Limit Theorem [8] can be invoked to justify drawing the $\theta_{i}$’s from a zero-average Gaussian distribution. Upon taking $\langle \theta_{2,i}^{2}\rangle =1$, we readily come to the traditional equation for the average squared distance that is diffused in a time interval Δt: $\langle \Delta x^{2}\rangle =2D\Delta t$.

The equation $\langle \Delta x^{2}\rangle =2D\Delta t$ does not contain a characteristic timescale. It is in order for the scale-free diffusion equation to ensue that we need to “adjust” the $\theta_{2,i}$’s and take $\xi_{2}\left(t_{i}\right)=\theta_{2,i}/\sqrt{\Delta t}$ in Equation (2).

In case of the 1D Lévy flight, we have for the stochastic ordinary-differential-equation and its discretized version, respectively:

(3) $$\dot{x}=\sigma \;\xi_{\alpha }\left(t\right)\;\;\mathrm{and}\;\;\Delta x_{i}=\sigma \;\theta_{\alpha ,i}\;\Delta t^{1/\alpha }.$$

Now the values for $\theta_{\alpha }$ are drawn from a symmetric, zero-centered α-stable distribution with a value of one for its scale factor. The Lévy flight is still scale-free, but because $\langle \theta_{\alpha ,i}^{2}\rangle \to \infty$ for 0<α<2, there is no longer a traditional diffusion equation and σ is a mere scale factor.

Figure 1 shows simulations of 2D random walks. At every timestep, a direction is chosen randomly from a flat distribution between zero and 2π. The displacement is the result of a random draw from a Gaussian distribution (Figure 1a) or from an α-stable distribution (Figure 1b). Both the Gaussian walk and Lévy walk are isotropic, i.e., taken from the center of the circle, all directions are equivalent. A generalization to more than 2 dimensions is readily formulated and simulated. The random walks then occur inside a ball with a finite radius. Whenever the domain boundary is hit, the particle comes to a standstill. For α=2, the random walk is symmetric under time reversal. However, as was already mentioned in the Introduction, for 0<α<2 the time-reversal symmetry is broken. It is not hard to understand why this is the case. When the particle is followed in forward time, we will often see a Lévy jump that makes the particle come to a standstill at the domain boundary. More rare will be a large jump from the domain boundary into the interior. When a movie of the moving particle is played backward, it will be the other way round. The forward and backward played movie are distinguishable.

Figure 2 shows the position distribution that results after a many-step 1D simulation on −1≤x≤1 for α=0.8. For α=2, a flat distribution results. However for 0<α<2, the Lévy jumps that terminate at x=±1 and the decreased mobility there lead to an increased probability density near x=±1. The Langevin Equation, Equation (3), can be equivalently formulated as a fractional Fokker–Planck Equation for the evolution of a probability distribution, i.e., $\partial_{t}p\left(x,t\right)=\sigma^{\alpha }\partial_{x}^{\alpha }p\left(x,t\right)$. The stationary distribution is then obtained as the solution of the ordinary differential equation that results when the left hand side is set equal to zero. The fractional derivatives are nontrivial, but in Ref. [23], a solution for the 1D case is presented:

(4) $$p_{st}\left(x\right)=\frac{2^{1-\alpha }\Gamma \left(\alpha \right)}{\Gamma^{2}\left(\alpha /2\right)}{\left(1-x^{2}\right)}^{\alpha /2-1},$$

where Γ(.) denotes the gamma function. Figure 2 shows this solution together with the results of the Langevin simulation. In Appendix A, we show with symmetry arguments that the normalized ${\left(1-r^{2}\right)}^{\alpha /2-1}$-form generalizes to the *n*D case, with *r* being the distance from the center of the ball.

It is worth noting that the U-shaped function as in Equation (4) and Figure 2 has been encountered in other systems in stochastic dynamics. For α=1, the Lévy stable distribution is actually the Cauchy distribution, $p_{1}\left(\xi \right)=\left(1/\pi \right)\left(\sigma /\left(\sigma^{2}+\xi^{2}\right)\right)$. For α=1 and upon taking x=2u−1, Equation (4) turns into $p\left(u\right)=\left(1/\pi \right){\left(u\left(1-u\right)\right)}^{-1/2}$, where 0≤u≤1. This is called the arcsine distribution because the cumulative distribution yields an arcsine: $\int_{0}^{u}p\left(u^{\prime }\right)\;du^{\prime }=\left(2/\pi \right)\arcsin\sqrt{u}$. In 1939, it was the same Paul Lévy who derived that the arcsine distribution emerges in the following case [28]. Let a 1D Brownian walk of duration *t* start at x=0. Next look at the fraction of time that the Brownian particle spends on the positive semi-axis. It is found that these fractions follow an arcsine distribution. This is called the arcsine law. Recently it has been discovered that arcsine laws occur more generally [29]. Driven mesoscopic systems are obviously out-of-equilibrium, but also in such systems, an arcsine law results when one considers, for instance, fractions of time that a current stays above its average value. Arcsine laws in nonequilibrium setups is currently a much researched topic [30,31] and Equation (4) may be a manifestation of something deeper and more general.

## 3. Two Connected Semicircular Reservoirs

**Figure 3:**
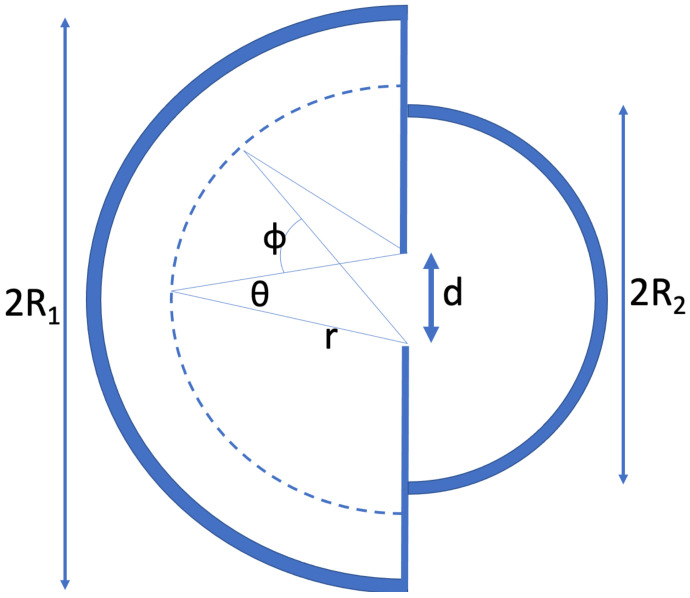
Two semicircular reservoirs with a small opening between them. The system contains a large number of noisy particles. At each timestep, each particle moves in an arbitrary direction with a displacement that is drawn from a Gaussian distribution or a Lévy-stable distribution as in Figure 1a,b. If a particle hits a semicircular wall, it comes to a standstill and only moves again if a computed displacement leads to motion inside the system. If a particle hits the straight vertical wall, it bounces elastically. For Gaussian noise, the system goes to an equilibrium with equal concentration on both sides of the opening. However, when the particles are subjected to Lévy noise, the steady state has an accumulation in the smaller reservoir.

Imagine a semicircular 2D domain with radius $R_{1}$ as in Figure 3. There is a small opening with a width *d* that gives access to a semicircular domain with radius $R_{2}$. We have $R_{2}<R_{1}$. Next imagine a large number of particles in this system. The particles are subjected to Lévy noise. In Appendix B, it is derived how there is a net flow into the smaller reservoir if both reservoirs have the same homogeneous particle density. Thus, when starting from thermodynamic equilibrium, a higher density develops in the smaller reservoir once Lévy noise starts being applied.

### 3.1. Distribution over the Two Reservoirs in Case of Lévy Noise

If the noise in the setup of Figure 3 is Gaussian, then the system will relax to an equilibrium with equal concentration in the two reservoirs. Each particle then has a probability $P_{1}=R_{1}^{2}/\left(R_{1}^{2}+R_{2}^{2}\right)$ to be in the larger reservoir and a probability $P_{2}=R_{2}^{2}/\left(R_{1}^{2}+R_{2}^{2}\right)$ to be in the smaller reservoir. The probability to be in a certain reservoir is in that case, simply proportional to the volume of that reservoir. In 2D, the “volume” is the area $V_{i}=\pi R_{i}^{2}/2$.

Next consider Lévy particles. The distribution will now be different. As was shown in the previous section and in Appendix A, Lévy particles tend to accumulate near the walls and in the smaller “nooks and corners”. With Lévy particles, the probability to be in the smaller reservoir will be larger than the reservoir’s fraction of the total volume.

For a stochastic simulation, we let the semicircular walls be “sticky” again, i.e., the particle comes to a standstill upon hitting the wall and only displaces again if a subsequent computed step leads to motion inside the system. If the linear vertical wall in the middle is hit, an elastic collision occurs. Thus, that wall is “bouncy”.

We will use the 2D solution for a circle, $p_{st}\left(r\right)=\left(\alpha /\left(2\pi \right)\right){\left(1-r^{2}\right)}^{\alpha /2-1}$, to come to an estimate of the steady-state distribution for the setup in Figure 3. We move to a description where $\rho_{i}\left(r_{i}\right)$, with i=1,2, denotes the normalized particle density in reservoir *i* at a distance $r_{i}$ from the opening. With:

(5) $$\rho_{i}\left(r_{i}\right)=\frac{\alpha }{\pi R_{i}^{2}}{\left(1-{\left(\frac{r_{i}}{R_{i}}\right)}^{2}\right)}^{\alpha /2-1}$$

it is readily verified that:

(6) $$\int_{r_{i}=0}^{R_{i}}\int_{\phi =-\pi /2}^{\pi /2}\rho_{i}\left(r_{i}\right)r_{i}\;dr_{i}\;d\phi =1.$$

With a large number of particles in the setup, there will be a relaxation to a distribution with a fraction $\varphi_{1}$ in reservoir 1 and a fraction $\varphi_{2}$ in reservoir 2. Obviously we have $\varphi_{1}+\varphi_{2}=1$. For any distribution over the two reservoirs we have:

(7) $$\rho \left(r_{1},r_{2}\right)=\varphi_{1}\rho_{1}\left(r_{1}\right)+\varphi_{2}\rho_{2}\left(r_{2}\right).$$

It is easy to see that $\int \int \rho (r_{1},r_{2})=1$, where the integration is over the entire 2-semicircle system in the figure.

The steady state occurs if there are as many 1→2 transitions as there are 2→1 transitions. We will next derive what values of $\varphi_{1}$ and $\varphi_{2}$ lead to steady state. In the above figure, imagine a semicircular strip of width $dr_{i}$ at a distance $r_{i}$ from the opening. The number of particles in the strip is $\rho_{i}\left(r_{i}\right)\pi r_{i}dr_{i}$ (i=1,2). We assume that for $r>r_{0}$, we are in the region where the power-law-description of the tail of the Lévy distribution ($p_{\alpha }\left(r\right)\propto r^{-(\alpha +1)}\;\mathrm{as}\;r\to \infty$) applies. The probability that the displacement during one timestep is *larger thanr* is then proportional to $r^{-\alpha }$. For small *d* and sufficiently large $r_{0}$, the angle θ, cf. Figure 3, will be small and we have d=θr. For a Lévy jump to lead to a particle transiting to the other reservoir, the jump must also be in the right direction. This brings in a factor (d/r)cosϕ, where ϕ is the indicated angle of the position on the semicircle with the horizontal. Integrating over ϕ from −π/2 to π/2, the full direction factor is found to be 2d/r. All in all, during one timestep we have for the number of cross-reservoir transitions from a distance between *r* and dr:

(8) $$dn_{i}^{tr}\left(r_{i},r_{i}+dr_{i}\right)\propto \varphi_{i}\frac{d}{r_{i}}r_{i}^{-\alpha }\rho_{i}\left(r_{i}\right)r_{i}\;dr_{i}.$$

Integrating from $r_{0}$ to the boundary $R_{i}$, we obtain for the number of Lévy-jump-associated transitions from reservoir *i*:

(9) $$N_{i}^{tr}\propto \frac{\varphi_{i}}{R_{i}^{2}}\int_{r_{i}=r_{0}}^{R_{i}}r_{i}^{-\alpha }{\left(1-{\left(\frac{r_{i}}{R_{i}}\right)}^{2}\right)}^{\alpha /2-1}\;dr_{i}.$$

The proportionality constant (associated with the ∝) and the $r_{0}$ (the radius from which the power law is taken to describe the Lévy-stable distribution) are the same for both reservoirs. At this point, it is also important to realize that for the Lévy jumps to dominate the number of 1→2 and 2→1 transitions, $R_{1}$ and $R_{2}$ must both be much larger than $r_{0}$.

*Mathematica* will readily give an analytical solution for the integral Equation (9). The solution involves the hypergeometric function [32]. Before working out Equation (9) in its full generality, we make a further simplification that will not affect the solution too much: As $R_{i}\gg r_{0}$ for both i=1 and i=2, we take $r_{0}=0$ to be the lower limit of the integral. With 0<α<1, the integral will not diverge with $r_{i}\to 0$. Next, the all-important reservoir radius $R_{i}$ can be scaled out of the actual integral and incorporated in the prefactor:

(10) $$N_{i}^{tr}\propto \frac{\varphi_{i}}{R_{i}^{2}}R_{i}^{-\alpha }R_{i}\int_{r_{i}=0}^{R_{i}}{\left(\frac{r_{i}}{R_{i}}\right)}^{-\alpha }{\left(1-{\left(\frac{r_{i}}{R_{i}}\right)}^{2}\right)}^{\alpha /2-1}\;d\left(\frac{r_{i}}{R_{i}}\right).$$

Upon taking $u=r_{i}/R_{i}$ and $v=u^{2}$ (so $dv=du^{2}=2u\;du$ and thus $du=1/(2\sqrt{v})\;dv$), further simplification is achieved:

(11) $$N_{i}^{tr}\propto \varphi_{i}R_{i}^{-1-\alpha }\int_{v=0}^{1}v^{-\alpha /2-1/2}{\left(1-v\right)}^{\alpha /2-1}\;dv.$$

The integral on the right-hand side is the well-known Euler integral, which is also known as the beta function [32]. Ultimately, this integral depends only on α. It is finite for 0<α<1 and as it is the same for both reservoirs, we find:

(12) $$N_{i}^{tr}\propto \varphi_{i}R_{i}^{-1-\alpha }.$$

The steady state condition is $\varphi_{1}R_{1}^{-1-\alpha }\approx \varphi_{2}R_{2}^{-1-\alpha }$. With $\varphi_{1}+\varphi_{2}=1$ we then get:

(13) $$\varphi_{1}\approx \frac{R_{1}^{1+\alpha }}{R_{1}^{1+\alpha }+R_{2}^{1+\alpha }},\;\;\varphi_{2}\approx \frac{R_{2}^{1+\alpha }}{R_{1}^{1+\alpha }+R_{2}^{1+\alpha }},\;\;\mathrm{and}\;\;\frac{\varphi_{1}}{\varphi_{2}}\approx {\left(\frac{R_{1}}{R_{2}}\right)}^{1+\alpha }.$$

**Figure 4:**
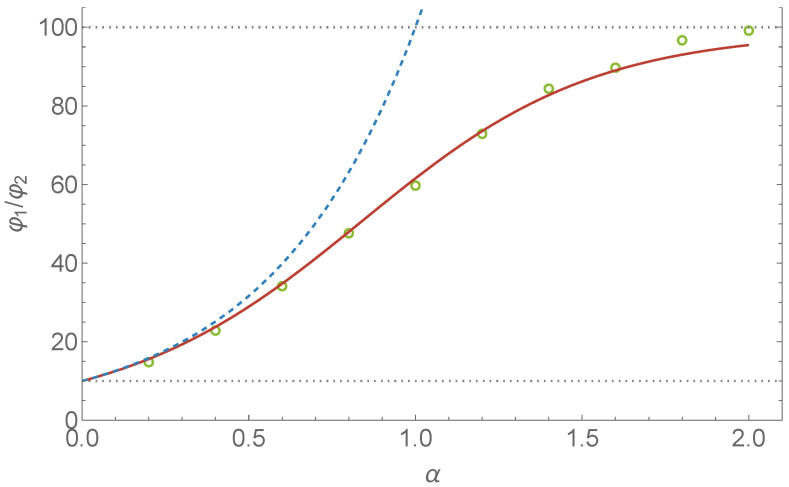
For the setup of Figure 3 with $R_{1}=10$ and $R_{2}=1$, we let $\varphi_{1}$ and $\varphi_{2}$ represent the fraction of particles in reservoir 1 and 2, respectively, at steady state. The curves depict the analytic approximations, Equation (13) (dashed) and Equation (15) (solid), of $\varphi_{1}/\varphi_{2}$. Each dot is the result of a stochastic simulation of 40,000 particles for $4\times 10^{5}$ timesteps (with Δt=0.001) following a $2\times 10^{5}$ timestep relaxation period. For the approximation according to Equation (15), we let $r_{0}=0.05$ and find good agreement with the result of the stochastic simulation.

**Figure 5:**
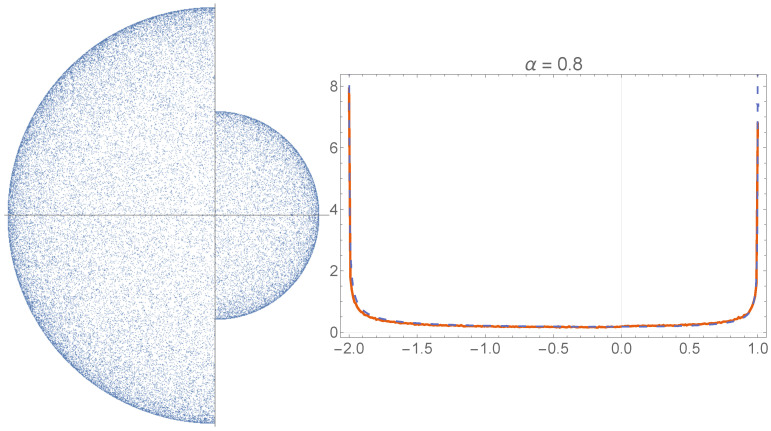
The figure on the left depicts a steady-state distribution for 50,000 Lévy particles in a two-reservoir confinement as depicted in Figure 3 after $10^{5}$ timesteps. We have $R_{1}=2$, $R_{2}=1$, and the opening has a width d=0.1. For the figure on the right we started with a steady-state distribution and ran the simulation for another $10^{5}$ iterations. We took a horizontal strip through the center with a width of 0.02 and partitioned it into 300 bins. Particles in each bin were counted and the results of the subsequent $10^{5}$ iterations were added. The solid line represents the resulting normalized 1D histogram. The dashed reference curve is the solution Equation (4). For the left reservoir, the domain was scaled to a length 2. Normalization of the combination of analytic solutions was done such that the probability to be in the left reservoir is 2/3. It is readily verified that this leads to continuity at the location of the opening.

The better approximation is obtained by not fully carrying through the $r_{0}=0$ simplification of the last paragraph. That the simple approximation according to Equation (13) fails for larger values of α is partly due to scaling issues. For the analytic approximation to be consistent with the numerics, we need $\Delta t^{1/\alpha }$ to be significantly smaller than $r_{0}$ (cf. Equation (3) with σ=1). Setting $r_{0}=0$ leads to a range where this is no longer true. As α becomes larger, this range becomes larger. Keeping $r_{0}>0$ in Equation (9), we find after some algebra and use of *Mathematica* for the equivalent expression of Equation (12):

(14) Nitr∝φiRi−1−α−πΓ(α2)Γ(3−α2)r01−αRi2F121−α2,1−α2;3−α2;r0Ri2,

where F12(a,b;c;z) is the aforementioned hypergeometric function. It is readily verified that the second term in the square brackets dominates for α→2 and small $r_{0}$. This is due to the $r_{0}^{1-\alpha }$ term. The hypergeometric function is defined as a power series [32] and under the $r_{0}\ll 1$ condition we can still take ${\left(r_{0}/R_{i}\right)}^{2}∼0$ and hence F12(.)≈1. The ratio of particles in the two reservoirs is then:

(15) $$\frac{\varphi_{1}}{\varphi_{2}}\approx {\left(\frac{R_{1}}{R_{2}}\right)}^{1+\alpha }\frac{\sqrt{\pi }\;R_{2}^{\alpha -1}-r_{0}^{\alpha -1}\Gamma \left(\frac{3-\alpha }{2}\right)\Gamma \left(\frac{\alpha }{2}\right)}{\sqrt{\pi }\;R_{1}^{\alpha -1}-r_{0}^{\alpha -1}\Gamma \left(\frac{3-\alpha }{2}\right)\Gamma \left(\frac{\alpha }{2}\right)},$$

which reduces to ${\left(R_{1}/R_{2}\right)}^{1+\alpha }$ (cf. Equation (13)) if we take α<1 and $r_{0}\to 0$ concurrently. Note, furthermore, that the equilibrium distribution, i.e., $\varphi_{1}/\varphi_{2}={\left(R_{1}/R_{2}\right)}^{2}$, is properly approached if we concurrently take α→2 and $r_{0}\to 0$. Both the approximations according to Equations (13) and (15) are depicted in Figure 4 and compared with the results of a stochastic simulation. Finally, it is worth pointing out that Equation (15) is still an approximation. The power law, Equation (1), that characterizes the Lévy-stable distribution is not valid for small values of ξ. For values of ξ near zero, the distribution is Gaussian-like and this is what is relevant for the behavior of particles close to the opening, i.e., r→0. Gaussian diffusion near the opening will lead to a continuous and differentiable steady-state concentration profile near the opening. This is also what Figure 5 shows.

Figure 4 shows the ratio $\varphi_{1}/\varphi_{2}$ as a function of α and compares the result of a stochastic simulation with Equations (13) and (15). We took $R_{1}=10$ and $R_{2}=100$. For α→0, the simple approximation according to Equation (13) leads to $\varphi_{1}/\varphi_{2}=10$. For the more sophisticated approximation according to Equation (15), the $\varphi_{1}/\varphi_{2}$ value at α→0 can be brought arbitrarily close to 10 by taking $R_{1}$ and $R_{2}$ much larger than $r_{0}$. There is 10 times as much “sticky wall” in the large reservoir and this result tells us that for α→0, all particles are concentrated at the sticky walls as would intuitively be expected.

The result that is derived in Appendix B hints at the reason that α=1 is “almost like” α=2. As we move away from the opening, the probability to hit the opening *decreases* as $r^{-\alpha }$. However, with a homogeneous distribution of particles, the number of particles at a distance between *r* and r+dr *increases* proportional to *r*. For an *n*-dimensional setup, the increase is proportional to $r^{n-1}$ (for n=2 we have circular strips and for n=3 we have spherical shells). All in all, we find that the number of “hits” from a distance *r* is proportional to $r^{n-\alpha -1}$. Note that for n=3, the entire range of α leads to an increase of “hits” with *r*. We have not done any further investigation of the 3D case. We see that for n=2, an increase of “hits” with *r* only occurs if α<1. For 1<α<2, the number of “hits” decreases with *r* and in that case transitions mostly happen from the region around the opening. This decrease with 1<α<2 also means that the particle exchange through the opening does not “feel” the different radii of the different reservoirs anymore.

Equation (4) describes and Figure 5 shows a nonhomogeneous distribution: As we move away from the opening, the concentration actually increases. This should add to the exponent n−α−1 that we derived in a previous paragraph. Some of this effect is incorporated in the approximation that led to Equation (15). Both that approximation and the simulations show an asymptotic approach to ${\left(R_{1}/R_{2}\right)}^{2}$ as α→2 and $r_{0}\to 0$.

### 3.2. Entropies and Energies Associated with Lévy Noise

The nonhomogeneous distributions shown in Figure 2 and Figure 5 essentially function as dissipative structures [27]. The depicted nonhomogeneous steady-state distributions represent a lower entropy than homogeneous distributions. However, these lower-entropy structures facilitate the transfer and dissipation of energy at steady state. The transferred energy comes in through the non-thermal motion of the active particles. It is next dissipated and released. Ultimately the low-entropy dissipative structures help the energy throughput and the entropy production.

As a result of the divergent standard deviation of the α-stable noise, the energy that is dissipated per unit of time is in principle infinite. The finite container size, however, truncates the Lévy jumps and make the aforementioned standard deviation of the jump sizes finite. We will not elaborate on this. What we will instead focus on in this subsection is the entropy decrease that is associated with the apparent nonhomogeneous distribution shown in Figure 5.

Imagine that the steady flow of energy that maintains the dissipative structure is suddenly halted. Such halting is straightforward if the active-particle-motion is, for instance, driven by magnetic forces or by optics. The distribution in Figure 5 will then homogenize. Such homogenization implies an increase in entropy and a concurrent decrease in free energy. Below we will find remarkably concise analytic expressions for the entropy change.

The relaxation towards homogeneity is two-part. First there is an intra-reservoir relaxation inside each of the two reservoirs to a spatially homogeneous spread. Next there is the slower relaxation between the two reservoirs towards a ratio $\varphi_{1}/\varphi_{2}=V_{1}/V_{2}=R_{1}^{2}/R_{2}^{2}$.

The entropy change associated with the intra-reservoir relaxations is hard to compute for the semicircular reservoirs of Figure 3 and Figure 5. However, for a circular reservoir as in Figure 1, it is easier and no resort to numerics is necessary. We take $p_{ini}\left(r\right)=\left(\alpha /2\pi \right){\left(1-r^{2}\right)}^{\alpha /2-1}$ as the initial distribution and $p_{fin}\left(r\right)=1/\pi$ as the final homogeneous distribution. It is well known that for a discrete set of probabilities, $p_{i}$, the associated entropy is given by $S=-\Sigma_{i}p_{i}\log p_{i}$. However, this summation cannot be straightforwardly extended to an integral for the case of a continuous probability density p(r). An obvious issue is that density is not dimensionless and that a logarithm can only be taken of a dimensionless quantity. In Ref. [33], it is explained how a sensible definition is only obtained after introducing another probability density that functions as a measure. We then obtain what is known as the relative entropy or Kullback–Leibler divergence [24]:

(16) $$D_{\mathrm{KL}}\left(p_{fin}||p_{ini}\right)=\int_{r\leq 1}p_{fin}\left(r\right)\log\left(\frac{p_{fin}\left(r\right)}{p_{ini}\left(r\right)}\right)dr.$$

When working out this integral, it is important to realize that the integration is from r=0 to r=1 over the area of a circle and that a term 2πr needs also be included. With the above expressions for $p_{ini}\left(r\right)$ and $p_{fin}\left(r\right)$, we find after some algebra that $D_{\mathrm{KL}}\left(p_{fin}||p_{ini}\right)=-1+\alpha /2+\log\left(\alpha /2\right)$. No such easy analytic solution ensues for more than two dimensions or even in the 1D case. The Kullback–Leibler divergence can be thought of as a kind of distance between two probability densities. However, it is generally not symmetric in the two involved distributions. In our case, we find $D_{\mathrm{KL}}\left(p_{ini}||p_{fin}\right)=-1+2/\alpha +\log\left(2/\alpha \right)$. Both $D_{\mathrm{KL}}\left(p_{fin}||p_{ini}\right)$ and $D_{\mathrm{KL}}\left(p_{ini}||p_{fin}\right)$ are remarkably simple expressions; they are continuous and concave up as α increases and reduce to zero for α=2.

The speed of the inter-reservoir relaxation depends on the size of the opening. For the small opening that is necessary for our approximations to be accurate, it will generally be slower than the intra-reservoir relaxation. For the inter-reservoir relaxation, the basic quantity is the probability to be in either of the two reservoirs. We go back to the basics to calculate what the entropy is for a given distribution over the two reservoirs.

In the Statistical Physics context, entropy is commonly defined as proportional to the logarithm of the number of microstates [1]. Imagine that there are *N* identical particles in the setup of Figure 3 and Figure 5. Here *N* is taken to be very large. In case of equilibrium, the number of particles in a reservoir is proportional to the volume $V_{i}=\pi R_{i}^{2}/2$ of a reservoir. With $\varphi_{i}N$ identical particles in reservoir *i*, the number of microstates in each of the two reservoirs is given by:

(17) $$\Omega_{i}=\frac{V_{i}^{\varphi_{i}N}}{\left(\varphi_{i}N\right)!}.$$

The numerator has the $\varphi_{i}N$-exponent because it is for each particle that the number of microstates is proportional to the volume. The microstate is the same, however, when two or more particles are exchanged. The denominator takes this into account and denotes the number of permutations among $\varphi_{i}N$ particles. With the definition S=logΩ and using Stirling’s approximation [1] (logN!=NlogN, if *N* is very large), we derive:

(18) $$S_{i}=\varphi_{i}N\log\left(\frac{V_{i}}{\varphi_{i}N}\right),$$

where “log” denotes the natural logarithm. As was mentioned before, at thermodynamic equilibrium the fraction of particles in a reservoir is proportional to the volume of that reservoir, i.e., $\varphi_{i}\propto V_{i}$. The argument of the logarithm in Equation (18) is then the same constant for both reservoirs. This leads to $S_{i}\propto \varphi_{i}$, as should be expected from an equilibrium-thermodynamics perspective.

We take for the total volume and the total entropy $V_{tot}=V_{1}+V_{2}$ and $S_{tot}=S_{1}+S_{2}$, respectively. It is next derived from Equation (18) that $S_{tot}=N(\varphi_{1}\log\left(V_{1}/\varphi_{1}\right)+\varphi_{2}\log\left(V_{2}/\varphi_{2}\right))-N\log N$. The additive NlogN-term is the same for all values of α. As it is only differences in entropy that matter, we discard this term. For the entropy per particle, $s_{tot}=S_{tot}/N$, it is next found:

(19) $$s_{tot}=\varphi_{1}\log\left(\frac{V_{1}}{\varphi_{1}}\right)+\varphi_{2}\log\left(\frac{V_{2}}{\varphi_{2}}\right).$$

**Figure 6:**
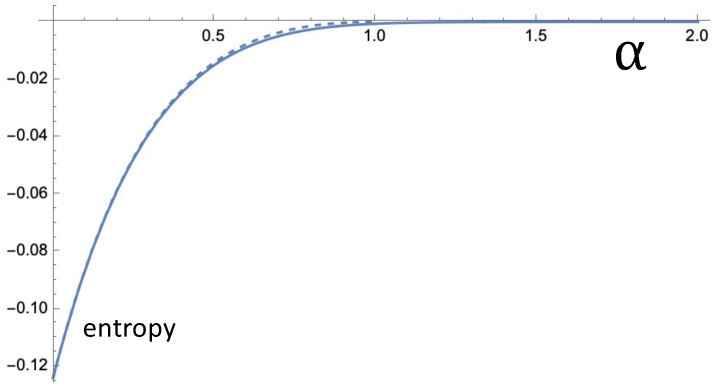
Given the setup of Figure 3 with $V_{tot}=1$ and $R_{1}=10R_{2}$, the curves show the entropy per particle, $s_{tot}$, as a function of the stability parameter α of the Lévy noise. The nonequilibrium noise leads to a concentration difference between the two reservoirs. The associated entropy decrease $s_{tot}$ is obtained by substituting into Equation (19) the approximate ratio according to Equation (13) (dashed curve) and according to Equation (15) (solid curve). For Equation (15) we took $r_{0}=0.05$, i.e., the value that led to good agreement with the stochastic stimulation (cf. Figure 4).

Figure 6 depicts $s_{tot}$ as a function of α following Equation (19). We took $V_{tot}=1$ (leading to $V_{1}=R_{1}^{2}/\left(R_{1}^{2}+R_{2}^{2}\right)$ and $V_{2}=R_{2}^{2}/\left(R_{1}^{2}+R_{2}^{2}\right)$) and $R_{1}=10R_{2}$. For the dashed curve, Equation (13) was used to come to the values of $\varphi_{1}$ and $\varphi_{2}$. For the solid curve the improved approximation, Equation (15) was used with $r_{0}=0.05$. The curves appear almost indistinguishably close. It is important to realize that this entropy also represents free energy. The free energy release associated with the equilibration can be obtained by multiplying the entropy (cf. Equation (19)) with the temperature. Again we emphasize that Equation (19) is related to just the inter-reservoir relaxation and does not incorporate intra-reservoir relaxation.

There is more thermodynamic way to derive the right-hand side of Equation (19) as the energy per particle that is invested in the building of the dissipative structure. With intra-reservoir equilibrium established, the chemical potential μ that is driving flux through the opening is the logarithm of the concentration ratio [1]. If we let ϕ be the fraction of the particles in the smaller reservoir, then we have $\mu \left(\phi \right)=\log\left(\frac{\phi }{V_{2}}\right)-\log\left(\frac{1-\phi }{V_{1}}\right)$. The energy that is dissipated when an infinitesimal fraction dϕ follows the potential and flows through the opening is μ(ϕ)dϕ. The entire equilibration takes ϕ from $\varphi_{2}$ to $V_{2}$. After some algebra and setting the temperature and the Boltzmann constant all equal to unity, it is found that the resulting total-equilibration-energy integral reduces to $-s_{tot}$ (cf. Equation (19)).

Equations (13) and (19) are concise and intuitive. Equation (13) is already a fairly accurate approximation. Given the geometry of the system and the value of α, Equation (13) gives the distribution over the two reservoirs. Equation (19) tells us what entropy decrease and what free energy “investment” is associated with the concentration difference between the reservoirs that gets established due to the active particle movement. It gives a measure for how far the system is driven from equilibrium by the active particle motion.

## 4. Discussion

In this article we explored a significant consequence of a bath in which particles velocities are Lévy-stable distributed. With the ordinary Gaussian velocity distribution that is associated with equilibrium systems, the maximization of entropy leads to particles homogeneously distributing in the confined domain. With a Lévy-stable distribution for the velocities, larger concentrations occur near the walls and in the smaller cavities. We have analytic expressions for the distribution of Lévy particles in the circular and the spherical domain. For the two connected reservoirs as depicted in Figure 3, we have derived a good approximation for the concentration difference between the reservoirs at steady state. We have presented an accounting of the entropies, and ensuing energies, for such divergence from equilibrium.

We have interpreted the nonhomogeneous particle distribution (cf. Figure 5) as a dissipative structure, i.e., a lower-entropy arrangement of particles that facilitates a larger dissipation of energy and concurrent larger production of entropy. There is nothing about heat conduction in the equations. However, it is tempting to hypothesize that with the particles being closer to the surface area, the system would be better able to transfer heat to the environment and do so at a larger rate.

In the 1990s, experiments were performed in which DNA, RNA, and proteins were manipulated on the molecular scale. This commonly involved breaking of molecular bonds. The involved energies were significantly larger than the $k_{B}T$ that can be considered as the quantum of thermal energy. In such far-from-equilibrium processes, Onsager’s reciprocal relations and other close-to-equilibrium concepts are no longer valid. Fortunately, at about the same time, theory was developed to handle fluctuations in far-from-equilibrium conditions. The Fluctuation Theorem [34] and the Jarzynski Equation [35,36] could very accurately account for the results of experiments in which microscopic beads were pulled by optical tweezers [37] and experiments in which RNA was forcibly unfolded [38,39]. However, it should be realized that the Fluctuation Theorem and the Jarzynski Equation apply when far-from-equilibrium events take place *in an equilibrium bath with a temperature*. In many experiments and real-life systems, the bath is the very *source of nonequilibrium*. The Fluctuation Theorem and the Jarzynski Equation are of no help in that case and new theory needs to be developed. An obstacle here is constituted by the fact that there is no equivalent of temperature for the Lévy-stable distribution of velocities that is commonly associated with the nonequilibrium bath. For a Gaussian velocity distribution, the standard deviation is proportional to the square root of the temperature. However for a Lévy-stable distribution, the standard deviation diverges and, technically, the temperature works out to be infinite. In this article we have tried to contribute to the development of description and understanding of what can happen in nonequilibrium baths.

As was explained in the Introduction, baths consisting of Lévy particles lead to similar physics as baths in which active particles are suspended. In both cases there is a continuous input of energy into the system and there is no longer a Fluctuation-Dissipation Theorem to guide the understanding and description. Swimming bacteria are a prime example of active particles. That swimming *Escherichia coli* bacteria can indeed be accumulated in cavities as has been experimentally demonstrated [40].

In a recent paper, results are presented of a numerical simulation of an active-particles-containing liquid [41]. A passive particle in this liquid was followed as a probe. This passive particle turned out to display Lévy-stable distributed displacements. What was simulated in this work was merely the Navier–Stokes equations and that passive particles exhibit these Lévy-stable distributed displacements is therefore a purely hydrodynamic effect due to active-particle-activity. That interesting and unexpected hydrodynamics can develop in liquids with immersed swimming bacteria has also been experimentally established [42].

The density profiles in Figure 2 and Figure 5 are mindful of the coffee ring effect. When a coffee drop on a surface evaporates, the stain that is left behind is darkest towards the edge [43]. This effect is common with liquids that carry solutes. There are technological applications where it is important to control the coffee ring effect. The simple explanation for the effect goes as follows. The drop has the shape of a disk. It has a fixed radius and the height of the drop vanishes near the edge. With a uniform evaporation across the surface area of the drop, there must be a net outward radial flow to replenish lost fluid near the contact line. Solute is carried along with this flow and ultimately deposited near the contact line. Much theoretical, numerical, and experimental research has been devoted to the effect in the last quarter century (see Ref. [44] and references therein). It is common to use equilibrium concepts like Einstein’s Fluctuation-Dissipation theorem when trying to account for the phenomenon. However, an evaporating drop is not in a thermodynamic equilibrium. It is certainly possible that solute particles exhibit the large jumps that are commonly encountered in nonequilibrium systems. The accumulation at the edge could then also be due to the mechanism that we discussed in this article.

In plasma physics, it is common to assume that the particles in a dense plasma follow the well-known Maxwell–Boltzmann distribution for particle speeds [1]. However, this equilibrium assumption may not always be valid, especially if the plasma is short lived and associated with an energy pulse. At Lawrence Livermore Lab, a table-top-size construction was developed to generate pulses of fast neutrons from high-energy deuterium collisions in plasma. Such collisions lead to the nuclear reaction D + D → ^3^He + n [45]. In the experiments, it appears that the number of produced neutrons exceeds the theoretical predictions by more than an order of magnitude. The reason for this is most likely that the Maxwell–Boltzmann distribution only applies at thermodynamic equilibrium.

Plasmas in which energy is converted or transferred are of course not in a thermodynamic equilibrium. In containers with plasma, a homogeneous distribution is therefore unlikely and accumulation at the edge as described in this work is possible. This is important because it means that fusion reactions in a plasma will occur at different rates at different positions. Through feedback mechanisms, such inhomogeneities may rapidly augment and possibly develop into serious instabilities.

Engineered microswimmers is probably the field where our results could ultimately be most applicable. There are good methods and technologies for manipulating suspended micrometer size particles from the outside with acoustic, magnetic, or optic signals (see e.g., Refs. [46,47]). Today the exciting new developments are in the medical application of such microrobots. Clinical uses for imaging, sensing, targeted drug delivery, microsurgery, and artificial insemination are envisaged and researched [48]. The microswimmers and microrobots are particles that are operating in a very noisy environment. Accumulations as described and explained in this article are likely to be encountered.

## Figures and Tables

**Figure A1:**
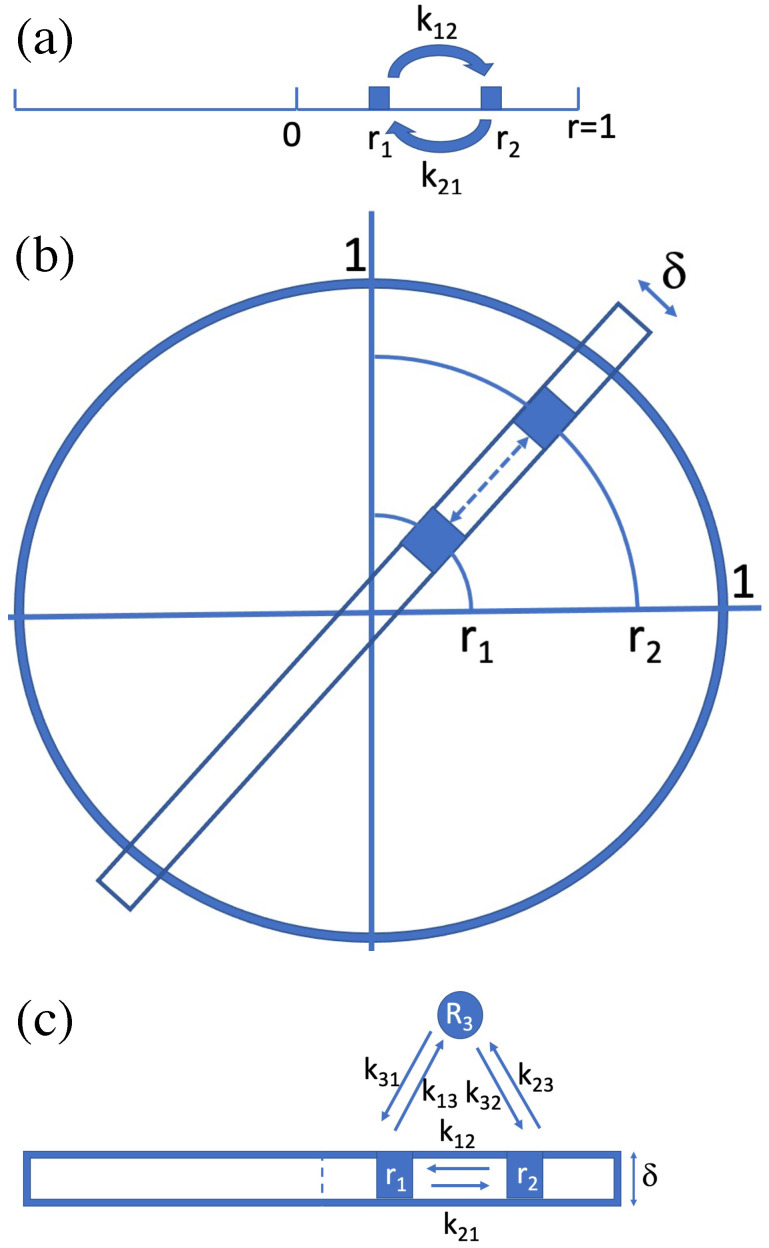
A Lévy walk in a confined domain. Whenever the particle hits the confinement wall, it comes to a standstill there. The 1D steady-state probability distribution (**a**) is solved in Ref. [23]: $p_{st}\left(r\right)\propto {\left(1-r^{2}\right)}^{\alpha /2-1}$. Between any two small intervals along the 1D domain, steady state implies $p\left(r_{1}\right)k_{12}=p\left(r_{2}\right)k_{21}$, where the *k*’s denote transition rates. In 2D (**b**) there is circular symmetry. If we take any narrow bar through the origin and look exclusively at traffic inside that bar, $p_{st}\left(r\right)\propto {\left(1-r^{2}\right)}^{\alpha /2-1}$ applies again. Next, we take a state $R_{3}$ outside the bar (**c**) and include transitions between $r_{1}$ and $r_{2}$ that go via any area $R_{3}$. As the circular symmetry implies the absence of vortices, transitions $k_{12}^{\prime }$ and $k_{21}^{\prime }$ that go via $R_{3}$ must also follow $p\left(r_{1}\right)k_{12}^{\prime }=p(r_{2})k_{21}^{\prime }$. From here it follows that $p_{st}\left(r\right)\propto {\left(1-r^{2}\right)}^{\alpha /2-1}$ also applies to higher dimensional setups. See the text of this Appendix for more detail.

## Appendix A. Extension of the 1D Solution to *n*D

Consider the steady-state solution $p\left(r\right)=C\left(\alpha \right){\left(1-r^{2}\right)}^{\alpha /2-1}$, where C(α) is the normalization factor (cf. Equation (4)). In this Appendix we will use symmetry arguments to show that this form generalizes to higher dimensional setups.

First consider the 1D ball depicted in Figure A1a and imagine a large number of particles distributed according to Equation (4). Next take two small intervals on the right side of $r=0:r_{1}<r<r_{1}+dr$ and $r_{2}<r<r_{2}+dr$, as depicted. At steady state and within any time interval Δt, there is as much flow from the $r_{1}$-interval to the $r_{2}$-interval as that there is from the $r_{2}$-interval to the $r_{1}$-interval, i.e., $J_{12}=J_{21}$. This is detailed balance [1]. Next we define a transition rate, $k_{12}$, that is the probability per unit of time for a particle in the $r_{1}$-interval to transit to the $r_{2}$-interval. The rate $k_{21}$ is analogously defined. Detailed balance implies that $k_{12}p\left(r_{1}\right)=k_{21}p\left(r_{2}\right)$ and thus:

(A1) $$\frac{k_{12}}{k_{21}}=\frac{p(r_{2})}{p(r_{1})}={\left(\frac{1-r_{2}^{2}}{1-r_{1}^{2}}\right)}^{\alpha /2-1}.$$

Next consider the 2D setup depicted in Figure A1b. A bar of width δ is going through the center of the circle. We take two little areas at distances $r_{1}$ and $r_{2}$ from the center. Consider only trajectories between these two areas that stay within the bar. The traffic inside the bar should mimic the 1D system that was considered in the previous paragraph and Figure A1a. Now consider also the transitions between the two little areas that proceed through trajectories that are not restricted to the narrow bar. Without loss of generality, we take an area $R_{3}$, cf. Figure A1c, and we consider trajectories between $r_{1}$ and $r_{2}$ that pass through $R_{3}$.

It is important to realize that the circular symmetry implies that there can be no vortices within the circular domain. Flow along any simple, closed curve within the unit circle would imply that there are points with net flow in the angular direction. Thus, along the $r_{1},r_{2},R_{3}$-loop there must be as much clockwise flow as there is counterclockwise, i.e., $J_{cw}=J_{ccw}$. This implies $k_{12}k_{23}k_{31}=k_{13}k_{32}k_{21}$ [49] and thus:

(A2) $$\frac{k_{12}}{k_{21}}=\frac{k_{13}k_{32}}{k_{23}k_{31}}.$$

The “state” $R_{3}$ can be taken to be anywhere within the circle and be of any size and shape. We can conclude that the ratio $k_{12}^{\prime }/k_{21}^{\prime }$ for transitions along any path between $r_{1}$ to $r_{2}$ within the circle must be equal to the ratio $k_{12}/k_{21}$ for transitions with trajectories inside the bar.

It follows that, for any dimensionality, the probability density at radius *r* must be proportional to ${\left(1-r^{2}\right)}^{\alpha /2-1}$. For a normalized probability density in *n* dimensions we derive:

(A3) $$p\left(n,r\right)=\frac{\Gamma \left(\frac{n+\alpha }{2}\right)}{\pi^{n/2}\Gamma \left(\frac{\alpha }{2}\right)}{\left(1-r^{2}\right)}^{\alpha /2-1}.$$

For n=2 the prefactor reduces to a simple α/(2π).

## Appendix B. Traffic between the Larger and the Smaller Semicircular Reservoir

Consider the setup in Figure 3 and Figure 5 and let a homogeneous particle density ρ be same in both reservoirs. In this Appendix we show that, if the particles are subject to Lévy noise, accumulation in the smaller reservoir develops. Imagine a semicircular strip of width dr at a distance *r* from the opening. There are ρπrdr particles in this strip. We let the power-law approximation, cf. Equation (1), be valid for $r>r_{0}$. Here $r_{0}$ is much larger than the width of the opening *d* and much smaller than the radii $R_{1}$ and $R_{2}$. The angle θ is small and with θ expressed in radians we have d=θr. For a particle in the semicircular strip at distance $r>r_{0}$, there is a probability that a Lévy jump will bring it to the other reservoir. To achieve such transition, the jump needs to be larger than *r*. For such a jump, the probability is proportional to $r^{-\alpha }$. In order to go through the opening, the jump must also be in the right direction. This leads to a factor (d/r)cosϕ (cf. Figure 3). After the integration from ϕ=−π/2 to ϕ=π/2, we derive a “direction factor” of 2d/r, i.e., ∝1/r. Putting together all of the effects specified in this paragraph, we have the following formula for the number of transitions during a small timestep from a distance between *r* and r+dr:

(A4) $$dn^{tr}\left(r,r+dr\right)\propto \frac{1}{r}r^{-\alpha }r\;dr=r^{-\alpha }\;dr.$$

Next we integrate from $r_{0}$ to the boundary $R_{i}$ (i=1,2) and find for the number of Lévy-jump-associated transitions from reservoir *i*:

(A5) $$N_{i}^{tr}\propto \int_{r_{0}}^{R_{i}}r^{-\alpha }\;dr\propto \mathrm{sgn}\left(1-\alpha \right)\left(R_{i}^{1-\alpha }-r_{0}^{1-\alpha }\right).$$

Care must be taken in case of α=1. In that case $N_{i}^{tr,\alpha =1}\propto \log R_{i}-\log r_{0}$. We thus conclude that for 0<α≤1, the number $N_{i}^{tr}$ diverges as $R_{i}$ is taken to infinity. For 1<α<2, a constant value for $N_{i}^{tr}$ ensues if $R_{i}\to \infty$.

The proportionality constant (associated with the ∝) and $r_{0}$ (the radius from which the power law is taken to describe the Lévy distribution) are the same for both reservoirs. Thus, if both reservoirs in Figure 3 and Figure 5 have the same uniform ρ, then we find for the net number of particles $\Delta N^{tr}=N_{1}^{tr}-N_{2}^{tr}$ that transits from the larger to the smaller reservoir:

(A6) $$\Delta N^{tr}\propto \mathrm{sgn}\left(1-\alpha \right)\left(R_{1}^{1-\alpha }-R_{2}^{1-\alpha }\right).$$

If 0<α≤1 and if values for $R_{1}$ and $R_{2}$ are large, then there is accumulation in the smaller reservoir.

For 1<α<2, there will again be accumulation in the smaller reservoir, but the effect becomes smaller as $R_{2}$ and $R_{1}$ grow and will become negligible as $R_{1,2}\to \infty$. Effectively, the geometry of the reservoirs is irrelevant for large $R_{1}$ and $R_{2}$. In that case it is particles near the opening that dominate the traffic through the opening.

In the main text, the above derivation is carried out for the case of a density $\rho_{i}\left(r_{i}\right)$ (i=1,2) that depends on the distance $r_{i}$ from the opening.

Finally, it is worth pointing out that the above derivation readily generalizes to higher dimensional reservoirs. In the 3D case, we face hemispheres. The number of particles in a hemispheric shell is $\rho 2\pi r^{2}dr$. For the *n*D case, the shell contains a number of particles that is proportional to $r^{n-1}dr$. We thus have for $dn_{nD}^{tr}$:

(A7) $$dn_{nD}^{tr}\left(r,r+dr\right)\propto \frac{1}{r}r^{-\alpha }r^{n-1}\;dr=r^{n-2-\alpha }\;dr.$$

This leads to:

(A8) $$N_{i,nD}^{tr}\propto \int_{r_{0}}^{R_{i}}r^{n-2-\alpha }\;dr\propto \left(R_{i}^{n-1-\alpha }-r_{0}^{n-1-\alpha }\right).$$

and

(A9) $$\Delta N_{nD}^{tr}\propto \left(R_{1}^{n-1-\alpha }-R_{2}^{n-1-\alpha }\right).$$

This is an interesting result. For 3 and more dimensions, we do not need to discriminate between different ranges of α. Lévy noise with any α (0<α<2) will in that case lead to a significantly higher density in the smaller reservoir and the effect will be stronger for higher values of $R_{1,2}$.
